# A Comprehensive Review of Mathematical Modeling for Drying Processes of Fruits and Vegetables

**DOI:** 10.1155/2022/6195257

**Published:** 2022-07-21

**Authors:** Ferdusee Akter, Ripa Muhury, Afroza Sultana, Ujjwal Kumar Deb

**Affiliations:** ^1^Department of Mathematics, Chittagong University of Engineering & Technology, Chattogram 4349, Bangladesh; ^2^Department of Physical and Mathematical Sciences, Chittagong Veterinary and Animal Sciences University, Chattogram 4225, Bangladesh; ^3^Department of Food Processing and Engineering, Chittagong Veterinary and Animal Sciences University, Chattogram 4225, Bangladesh

## Abstract

Drying fruits and vegetables is a procedure of food preservation with simultaneous heat, mass, and momentum transfer, which increases the shelf life of the food product. The aim of this review was to provide an overview of the researches on mathematical modeling for drying of fruits and vegetables with the special emphasis on the computational approach. Various heat-mass transport models, their applications, and modern drying technologies to the food industry have been reported in this study. Computational fluid dynamics, a new approach for solving heat and mass transfer problems, increases the accuracy of the predicted values. To investigate the parameters of drying needs a significant amount of time as well as costly laboratory and experimental efforts. Therefore, computational modeling could be an effective alternative to experimental approaches. This review will be beneficial for future studies in drying processes, especially for modeling, analysis, design, and optimization of food science and food engineering.

## 1. Introduction

Micronutrient rich fruits and vegetables may assist to overcome malnutrition of individuals. In addition, they are rich in unsaturated fat, dietary fiber, and phytochemicals that are beneficial to health [1]. It is estimated that one-third of the world's food supply is lost owing to poor post-harvest processing practices every year [2]. In underdeveloped nations, such as Bangladesh, this loss is believed to be between 30 and 40 percent of the production [3]. This is seen as a major source of nutritional and economic harm for both individual countries and the whole planet. Because of this, it is essential to investigate and find the best techniques of food processing and preservation in order to reduce food losses [1].

Produce changes seasonally; so, it is impossible to have year-round access to a wide variety of fruits and vegetables. As a result, many procedures have been implemented in order to provide consumers with an alternate method of consuming and applying food [4]. Because of this, processing and preservation are essential in the fruit and vegetable supply cycle because they link agricultural output to the delivery of required [5].

When it comes to fruit and vegetable preservation, drying is the most common unit activity. Bacteria and mold thrive in fruit because of the high water content, which makes them ideal for growing. Remove the moisture from the food and prevent the formation of bacteria by drying the food [6, 7]. For the preservation of fruits and vegetables, hot air drying is the most often used drying technique. More than 85% of all industrial dryers are convective dryers, which use hot air or combustion gases to transfer heat [8]. It is common for dried food to have less than 25% water content and a water activity of less than 0.06 [9, 10]. Food drying may enhance storage capacity, reduce production needs, and decrease the weight and transportation costs of food [11]. The drying process not only helps to preserve the product but also has the potential to improve the overall quality of the materials and to produce value-added compounds. Moreover, a novel method or one that combines existing methods is required to ensure the excellent quality of the dried items [12–14]. The improved technologies diminish energy consumption by as much as 80% and improve drying efficiency by as much as 26.5% in terms of reduction in drying time [15]. In addition to this, the idea of quality needs to include both the energy efficiency and the effect that making sustainable dry goods has on the environment [13]. In order to simulate and verify the drying process, it is important to employ mathematical models. Through the use of virtual laboratories, it is possible to achieve results that would be impossible or impractical to obtain at the conceptual stage of research [16]. Mathematical modeling in food drying is the use of mathematical equations to predict the drying process's behavior [17]. Drying air velocity and temperature both have a significant impact on the drying rate, and the rate of evaporation may slow down as a result of moving the products. For quality and storage safety reasons, it was important to keep the air temperature low at the beginning and end of the process [18–20]. Mathematical models are helpful in optimizing the drying conditions and studying complicated heat and mass transport processes. Furthermore, drying conditions and simulation model parameters are linked. Experimental error is reduced due to the drying model, and the drying process is enhanced while energy consumption is reduced and profit margins are increased as a result [21, 22]. The goal of this study was to shed light on mathematical modeling and how it might be used to speed up the drying process for fresh produce. An investigation of fruit and vegetable drying model possibilities and future prospects was conducted. Future researchers in food science and food engineering will benefit from this study, especially those working on fruit and vegetable drying models, analyses, designs, and optimizations.

## 2. Main Text

### 2.1. Drying Process

**Figure 1:**
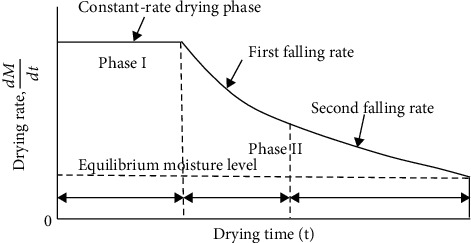
Drying rate curve [24].

The drying of fruits and vegetables is a sophisticated process that includes interconnected transitory processes of heat, mass, and momentum transfer, as well as physical, chemical, and phase changes [23]. There are two drying regimes when food items are heated, as indicated in Figure 1. Phase I begins with the evaporation of surface moisture. This process continues until enough water is available at the surface to warrant evaporation. Surface area and temperature are both important in phase I because they affect the reaction's first phase. However, phase II is all about the transport of moisture from the inside to the outside and the subsequent evaporation of that water. For a short time, there is a slowdown in drying, and it is done when there is an equal amount of air and solid vapor pressure in the surrounding air [24].

### 2.2. Classification of Fruits and Vegetables Drying Model

**Figure 2:**
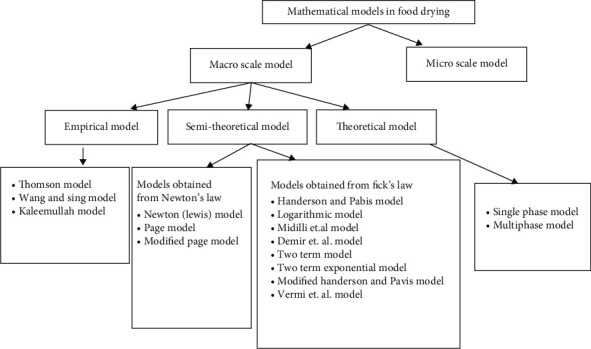
Classification of mathematical models in food drying [6, 26, 29–31].

There were two scales used in the mathematical models, a macroscale (10^−3^–10^0^ m) model and a microscale (10^−6^–10^−3^ m) model. A multiscale model is a model that incorporates both of these features [25–27]. Microscale models provide more accurate predictions of transport processes and deformation in food material throughout the drying process than macroscale models do at the cellular level [26]. Process design, optimization, energy integration, and control are all made possible via the use of effective models. As a result, the mathematical models and their applications in forecasting the drying kinetics of food should be developed and refined on a daily basis [16, 28]. The classification of food drying models is shown in Figure 2.

### 2.3. Theoretical Models and Their Applications

In physical-based modeling, theoretical models of the drying process are determined by mathematical deduction. In this section, we build a boundary value problem by taking into account the governing equations of transport phenomena, assumptions, and starting and boundary conditions. The boundary value issue is then solved analytically or numerically, and the results are compared to those obtained experimentally. Increase the drying temperature from 313 to 353 degrees Celsius, and you will save around 40% of the drying time, according to research [32]. Furthermore, with constant and varied surface transfer coefficients, the distribution of moisture did not change any more than before [33]. Tables 1 and Tables 2 provide the governing equations and boundary conditions, respectively, which are utilized in the food sector for modeling to dry products and ingredients.

Even in the absence of experimental evidence, a physics-based model accurately predicts the occurrence of physical phenomena. A variety of theoretical models, as well as their implementations on diverse fruits and vegetables, are shown in Table 3.

### 2.4. Solution Processes of the Theoretical Model

Analytical and numerical methods can be used to solve the partial differential equations. The governing equations of the drying processes are complex, and so it is difficult to find the solution both analytically and numerically. Therefore, we must make assumptions to simplify the mathematical model so that it can be solved for an exact or numerical solution, but the analytical technique is most commonly employed to solve the uncoupled mass transfer equation in one dimension. The governing equations are rewritten using the separation of variables approach and dimensionless Fourier and Biot variables [46].

Moreover, fruits and vegetables are naturally three-dimensional, and hence three-dimensional modeling provides a more accurate representation. Numerous types of studies have been published during the previous decades; although, the majority of models are two-dimensional. The primary benefit of the two-dimensional model is that it can be implemented with more precision and at a lower cost of computing [26]. At the moment, the three most often utilized techniques for discovering high-accuracy approximation solutions to transport phenomena are finite difference, finite volume, and finite element methods [28, 43].

The finite difference approach can only be used to solve issues with simple geometrical structures. The differential terms are decomposed into discrete representations via domain discretization on a rectangular grid for the finite differences. Due to the variable geometry of fruits and vegetables, this approach is typically used in rectangular areas, which restricts its usefulness [47].

One of the most common numerical methods is the finite volume method. A variety of polyhedra, some of which are not regular grids, are generated as a result of this method of discretizing the domain. In comparison to the finite-difference technique, the usage of polyhedron grids with integral approximations has several advantages. It supports various domain geometries, making it easier to use in multiple dimensions [48].

The finite element approach is more adaptable and broad. Besides, the approximation approach differs from that of the finite volume method. The weak formulation of the model is required for algorithm creation. Test functions must also be used in order to apply the Gauss theorem, which is typically connected with mass, energy, and momentum conservation rules. The finite element method gives a better approximation than other methods for complex geometries. So, the finite element method is the one most applied by researchers [49, 50]. Numerous software programs utilize numerical methods to solve mathematical models, and the answer is referred to as numerical simulations.

#### 2.4.1. Computational Approach

**Figure 3:**
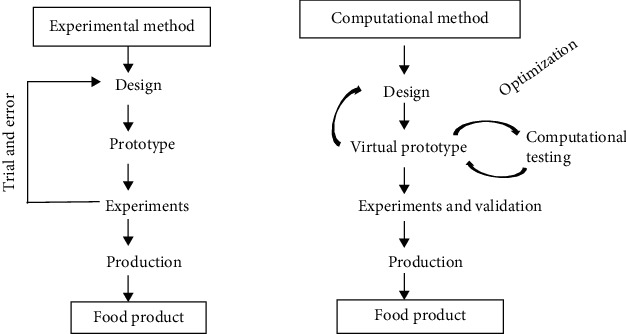
Experimental and computational methods applied to food product [51].

Computational fluid dynamics (CFD) is currently a critical instrument for solving issues in science, engineering, and the food business, and it is the most widely used and well-known modeling approach based on numerical methods (Figure 3). Through CFD simulation, it is possible to predict the transport phenomena during the drying process, the nutritional value, and food processing and storage conditions, reducing the need for trial and error in experimental techniques [51].

CFD analysis relies heavily on mesh creation. In the past few years, meshing technology has come a long way, and the use of tetrahedral, hexahedral hybrid, and polyhedral meshes has helped get around the problems with simpler meshes [52].

**Figure 4:**
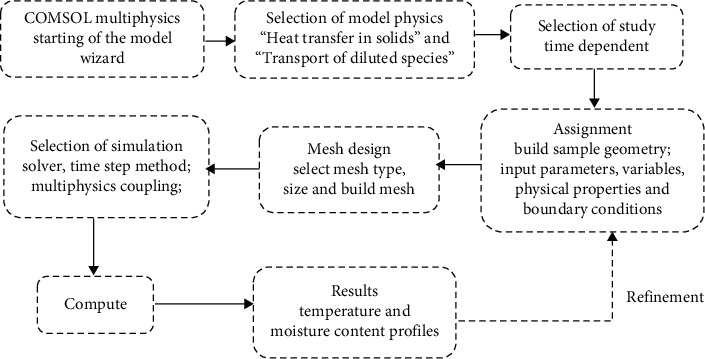
Flow chart for implementation of the coupled heat and mass transfer model and simulation procedure using COMSOL [12, 39].

A number of commercial CFD software such as COMSOL, ANSYS, MATLAB, and FORTRAN, among others, are widely used to simulate the model of drying processes [23, 43, 53, 54]. Employing response surface methodology (RSM), the drying behavior of fruits and vegetables, was optimized and different process factors were studied [55–58]. An understanding of the transport phenomena that take place during drying as well as better management of the drying process is possible via the use of these approaches [59]. Simulation procedure of the drying process using COMSOL Multiphysics is shown in Figure 4.

### 2.5. New Technologies of Modeling Fruits and Vegetables Drying

**Figure 5:**
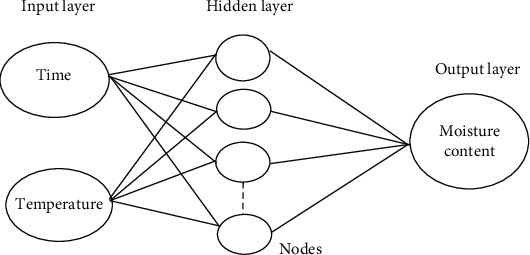
ANN structure [61].

The artificial neural network (ANN) is a new way of modeling in the food industry that is very important to the development of new technologies. An artificial neural network is made up of a huge number of interconnected processing units. There are three layers in an ANN: input, hidden, and output. Neurons or nodes make up the bulk of an ANN's structure (Figure 5). Problems that standard statistical and mathematics approaches fail to solve can be solved with the nonlinear statistical methodology known as artificial neural networks [60].

Medical and technical fields alike may benefit from ANN's data-driven power. There are several applications for this in food processing such as the processing of fruits like mango, banana, and pomegranate, as well as the processing of berries like mulberry and strawberry, as well as dates [61, 62]. The drying kinetics are properly predicted by the ANN model [63]. Furthermore, there have been studies using this technique for modeling the color and phenolic content of apples as well as the antioxidant activity of bananas [64, 65]. The antioxidant activity and phenolic components of bananas under various drying processes were modeled using an artificial neural network by Guine et al. [65]. In comparison to other fresh fruits, bananas in the dry source were shown to have lower concentrations of phenolic compounds and antioxidant activity. They used basic ANNs to show that the quantities of phenolic compounds and antioxidant activity may be projected with high accuracy from the drying condition, banana variety, and the specific kind. Onwude et al. found that the ANN model is able to provide more accurate results about the drying process of pumpkin in comparison to other kinetic models. Moreover, it is able to produce better outcomes even if the experimental settings and data sets are modified as a consequence of the incorporation of more experimental data [66].

Conventional drying techniques include sun drying, solar drying, hot air drying, spray drying, freeze drying, and vacuum drying. Sun drying uses heat energy directly from the sun, and a temperature of at least 86°F is required, with higher temperatures preferred. Sun drying might be risky due to the uncontrollable nature of the weather. Sun drying works best when the relative humidity is less than 60% [67]. On the other hand, foods may be dried using the heat energy from the sun in a particular dehydrator in a controlled way that not only raises the temperature but also enhances the airflow. This is known as solar drying. Many food drying applications can be completed successfully at temperatures below 60°C without risk of thermal damage. Because temperatures beyond 60°C are rarely attained in small-scale drying facilities, solar drying prevents exposing the food material to too high temperatures. This method speeds the drying process and lowers the chance of mold or spoilage in the food [67, 68]. Drying with hot air has several benefits, including a long shelf life, an easy design and operation, a low cost, and a simple cost structure, but it takes a long time to complete. Spray drying results in a product that is both excellent quality and has a low moisture content. It is utilized in the microencapsulation process, as well as the creation of powder, and instant powder but the installation cost is high. Freeze drying eliminates oxidation damage, minimizes changes to chemical components, reduces shrinkage, keeps the porosity structure of food, and is a very costly method [13].

Novel drying methods include microwave, infrared, radiofrequency, osmotic, supercritical, pulse electric field, and heat pump drying. Improved drying efficiency and product quality may be achieved by using these methods. There has been a slew of different drying approaches investigated in tandem. There are many ways to combine convection and microwaves, convection and osmotic dehydration, convection and infrared radiation, convection and ultraviolet radiation, and many other combinations [13, 61, 67, 69]. Drying in combination seems to be the most promising strategy, since it ensures high quality while using the least amount of energy. There is still room for improvement in the drying process, though. Food items with high health-promoting characteristics and attractive sensory features will be fortified with additional treatments to promote water diffusivity and retention of important chemicals [4]. The capacity to minimize costs, be environmentally friendly, and assure the great quality of the dried objects are the most significant factors [13].

### 2.6. Recommendations for Future Research

The enhancements of some models could be done in the upcoming years:

- 3D modeling of fruits and vegetables
- Give emphasize on microscale modeling and multiscale modeling
- Optimization of the process conditions
- Future research in the food drying model would consider new approaches such as multiphase models at the cellular level, multiphase multiscale models, transport models considering intracellular, and intercellular water separately [26]
- The application of deep learning and neurofuzzy system-based models in food processing research [62]
- Future research on organic foods in comparison to conventional products is required due to the growing demand for organic products [70]

As a consequence, it is needed to establish a relationship between process conditions, food structure, and product quality by researchers and specialists from different fields using their knowledge and resources.

## 3. Conclusions

Drying fruits and vegetables is a time-consuming and energy-intensive procedure that demands a great deal of energy and time. It extends the shelf life of the product while reducing its bulk and weight, which simplifies shipping. Dried foods are palatable, healthy, light, and convenient to transport. Modeling is a novel technique for evaluating experimental data that has gained prominence in recent years. Several forms of theoretical models with application to various fruits and vegetables and the methods of solution were discussed in this paper. Fluid flow, heat transfer, and product quality may all be predicted using CFD in a variety of drying systems. It is possible to overcome some of the disadvantages of macrobased modeling by using new tools and methodologies, such as multiscale or micromodeling. Moreover, the ANN model can properly predict the drying kinetics, color changes, phenolic compounds, and antioxidant properties in various drying processes. Conventional methods are less expensive, easier to use, and take longer drying time. However, novel drying techniques ensure the quality of food using low energy consumption. There are advantages and disadvantages to each process, but the most important for a newly developed technology is the ability to minimize costs, be ecofriendly, and provide excellent functional, physical, and sensory quality in the dried items. The models discussed here might readily be extended to other agricultural products with complicated structures and comparable heat and mass transfer processes, such as cooking, frying, or roasting. Environmentally friendly technology is becoming more prevalent in today's society, and every advancement in this field is highly commended. These breakthroughs need a tremendous amount of time and money spent in laboratories and on experiments. Mathematical models and computer simulation tools might be used in place of experimental procedures with success.

## Figures and Tables

**Table 1:** Transport equations during drying of fruits and vegetables.

Model type	Governing equations	References
Macroscale	Heat transfer equations	
	1. *ρc*_*p*_(*∂T*/*∂t*) + *ρc*_*p*_∇*T* = (*k*∇*T*) + *Q*_inf_	[12]
	2. *ρc*_*p*_(*∂T*/*∂t*) = ∇·(*k*∇*T*)	[18, 34–36]
	3. *ρC*_*p*_(*∂T*/*∂t*) + ∇·(−*k*∇*T*) + *ρC*_*p*_ *u* · ∇*T* = 0	[23, 33]
	4. *ρ*(*T*)*c*_*p*_(*T*)(*∂T*/*∂t*) = (*∂*/*∂x*)(*k*(*T*)(*∂T*/*∂x*)) + (*∂*/*∂y*)(*k*(*T*)(*∂T*/*∂y*)) + (*∂*/*∂z*)(*k*(*T*)(*∂T*/*∂z*))	[37]
	(5) *∂*(*ρc*_*p*_*T*)/*∂t* = 1/*r*{(*∂*/*∂r*)(*k* *r*(*∂T*/*∂r*)) + (*∂*/*∂z*)(*k* *r*(*∂T*/*∂z*))}	[38]
	Mass transfer equations	
	6. (*∂* *c*/*∂* *t*) + ∇·(−*D*_effs_∇*c*) = 0	[12, 35]
	7. *∂c*/*∂t* = ∇·(*D*∇*c*)	[33, 34]
	8. *∂*(*ρc*)/*∂t* = 1/*r*{(*∂*/*∂r*)(*D*_eff_*ρ* *r*(*∂* *c*/*∂r*)) + (*∂*/*∂z*)(*D*_ef*f*_*ρ* *r*(*∂* *c*/*∂z*))}	[38]
	9. $\begin{array}{c} \partial c/\partial t=D\left(1/r\left(\partial /\partial r\right)\left(r\left(\partial c/\partial r\right)\right)\right)+D\left(1/r\left(\partial^{2}c/\partial \phi^{2}\right)\right)+D\left(\partial^{2}c/\partial z^{2}\right)\\ r_{i}\leq r\leq r_{0};0\leq \phi \leq 2\pi ;0\leq z\leq 1 \end{array}$	[39]
	10. (*∂c*/*∂t*) + ∇·(−*D*∇*c*) + *u* · ∇*c* = 0	[23, 40]
Microscale	Heat transfer equation 11. *ρ*_*c*_*c*_*p* *c*_(*∂T*/*∂t*) + *ρ*_*c*_*c*_*p* *c*_∇*T* = ∇·(*k*_*w*_∇*T*)	[41]
	Mass transfer equation 12. (*∂c*/*∂t*) + ∇·(*D*_*c*_∇*c*) = 0	[41]

**Table 2:** Initial and boundary conditions.

Transport phenomena	Initial and boundary equations	Transfer mechanism	References
Heat and mass at *t* = 0	T = T_0_ *c* = *c*_0_	**—**	[18, 38, 41–43]
Heat	*n* · (*k*∇*T*) = *h*_*T*_ (*T*_*a*_ − *T*_*s*_)	Convection	[33]
Heat and mass	*n* · (*k*∇*T*) = *h*_*T*_(*T*_*a*_ − *T*_*s*_) − *h*_*m*_*ρ*(*M* − *M*_*e*_)*h*_*fg*_	Convection, conduction, and diffusion	[12, 23, 37]
Mass transfer	*n* · (*D*∇*c*) = *h*_*m*_ (*c*_*b*_ − *c*)	Diffusion	[18, 33, 39]
Heat transfer at the symmetry/insulated boundary	*n* · (*k*∇*T*) = 0	Convection	[12, 23, 42]
Mass transfer at the symmetry/insulated boundary	*n* · (*D*∇*c*) = 0	Diffusion	[12, 23, 42]
Mass transfer cell to cell	*n* · (*D*_*w*_∇*c*) = *h*_wall_(*c*_mi_ − *c*_*i*_)	Diffusion	[41]
Mass transfer cell to intracellular space	n · (*D*_*w*_∇*c*) = *h*_wall_(*c*_mi_ − *c*_*a*_)	Diffusion	[41]

**Table 3:** Applications of theoretical models.

Material name	Geometry	Drying method	Outcomes
Sweet potato [12]	Cylindrical shaped, 2D axisymmetric	Infrared drying	The moisture content was found to be susceptible to a low and high effective diffusion value. Infrared drying alone has a number of drawbacks, which could be overcome by combining infrared heating with other drying techniques.
Papaya [37]	Rectangular shape	Convective drying	This model gives a better output of the phenomena inside the sample. It is reported that the moisture content is nonuniform in the fruit sample and differs in each location. The authors also observed that 3D models would be better than the 2D models in agreement with the experimental data.
Mango [35]	Mango slab 3D modeling	Solar drying	To solve the simultaneous heat and mass transport problem on the surface area of food drying, a simple numerical technique has been presented here in this paper. The approach relies on the water activity that was created from experimental data.
Sultana grapes [34]	Solid spherical	Computer controlled drying system	The kinetic model predicts the development of color in the product at each node during the drying process, whose response rate depends on the temperature and moisture content of the product, thereby coupling the changes in product color with local heat and mass transfer predictions. This involves separating excellent fruit from stems, cap, stems, gravel, and other foreign materials. Further processing can cause damage to the fruit's skin, allowing sugar to escape, making the fruit sticky.
Banana [38]	Cylindrical	Computer controlled dryer	The arbitrary Lagrangian-Eulerian (ALE) approach was implemented to incorporate the axial and radial shrinkage effects into the finite element model (FEM) model.
Mushroom [36]	2D axis symmetric	Cabinet air drying	A finite element model was developed to examine the temperature and moisture profiles inside the mushroom by including the phase shift during mushroom dehydration in a cabinet-air-dryer. To improve the transport process predictions, the model incorporates a variety of parameters, including heat transfer coefficient and mass transfer coefficient as well as the mushroom's water activity and specific heat.
Pineapple [42]	Rectangular 2D axisymmetric	Tray dryer	A comparison between the average volume of the test and the expected moisture ratio values for all drying settings was analyzed in this paper. These models are capable of predicting a suitable pattern for the moisture concentration profile.
Tomato slices [44]	Rectangular 2D axisymmetric	Tunnel dryer	According to this study, increased temperature and speed of air can also shorten drying time. Comparing the theoretical and experimental drying kinetics, the root mean square error is found to be about 8 percent.
Apple slice [18]	Square shape	Hot-air drying	To predict the hot-air drying process of apple slices, a heat-mass transfer combined with a stress-strain mathematical model was employed. The image processing technology was utilized to measure the drying shrinkage deformation of apple slices. Apple slices were more susceptible to drying shrinkage deformation due to moisture stress than thermal stress. The observed result was that moisture stress had a stronger influence on drying shrinkage deformation.
Apple [45]	Slab cylindrical	Intermittent microwave convective (IMCD) drying	The drying process of IMCD is significantly faster than the drying of convection. The essential basis of the model allows us to better understand the drying kinetics and the IMCD heat and mass transfer.
Pineapple [39]	3D ring shape	Laboratory scale hot air drying	The moisture characteristics are substantially influenced by the deformation that occurs during shrinking. As a result, when building a food drying model, shrinkage should not be overlooked.
Prune [23]		Convective air drying	The author found a good agreement with the predicted and experimental values of drying data with the coupled heat, mass, and momentum transfer model. It is hoped that this model would be used for other food processes and food products with related phenomena.
Granny smith apple [41]	Microscale	Convective drying	The temperature distribution was also predicted effectively in the cells and intercellular spaces. During drying, it was observed that the air-filled intercellular spaces were heated more rapidly than the cells.

## Abbreviations

ALE: Arbitrary Lagrangian-Eulerian
ANN: Artificial neural network
CFD: Computational fluid dynamics
FEM: Finite element method
IMCD: Intermittent microwave convective drying
*c*: Moisture concentration (mol/m^3^)
*c*_*b*_: Bulk moisture concentration (mol/m^3^)
*c*_*i*_: Water concentration of the adjacent cell (mol/m^3^)
*c*_mi_: Water concentration of the cell (mol/m^3^)
*c*_*p*_: Specific heat capacity of material (Jkg^−1^ K^−1^)
*D*: Moisture diffusivity (m^2^/s)
*D*_cw_: Diffusivity of the cell wall
*h*_fg_: Latent heat of evaporation (J/kg)
*h*_*m*_: Mass transfer coefficient (ms^−1^)
*h*_*T*_: Heat transfer coefficient (Wm^−2^ K^−1^)
*h*_wall_: Water transfer coefficient of the cell wall (ms^−1^)
*k*: Thermal conductivity (Wm^−1^ K^−1^)
*k*_*w*_: Thermal conductivity of cellular water (Wm^−1^ K^−1^)
*p*: Pressure (Pa)
*T*: Drying temperature (°C)
*t*: Drying time (s)
*T*_*s*_: Sample surface temperature (°C)
*Q*_inf_: Volumetric infrared heat source
*ρ*: Density of the sample (kg/m^3^)
*M*: Moisture content (kg/kg) db.

### Subscripts

0: Initial
*c*: cell
*e*_*ff*_: Food effective property.
